# Monocyte-Derived Dendritic Cells as Model to Evaluate Species Tropism of Mosquito-Borne Flaviviruses

**DOI:** 10.3389/fcimb.2019.00005

**Published:** 2019-01-28

**Authors:** Obdulio García-Nicolás, Marta Lewandowska, Meret E. Ricklin, Artur Summerfield

**Affiliations:** ^1^Institute of Virology and Immunology (IVI), Bern, Switzerland; ^2^Department of Infectious Diseases and Pathobiology, Vetsuisse Faculty, University of Bern, Bern, Switzerland; ^3^Graduate School for Cellular and Biomedical Sciences, University of Bern, Bern, Switzerland; ^4^Department of Emergency Medicine, Inselspital, University Hospital Bern, Bern, Switzerland

**Keywords:** Flavivirus, monocyte-derived dendritic cells, *in vitro* model, infection, tropism, innate immune response

## Abstract

Several mosquito-borne Flaviviruses such as Japanese encephalitis virus (JEV), West Nile virus (WNV), Dengue Virus (DENV), and Zika virus (ZIKV) can cause severe clinical disease. Being zoonotic, Flaviviruses infect a wide variety of terrestrial vertebrates, which dependent of the virus-host interactions, can enhance ongoing epidemics and maintain the virus in the environment for prolonged periods. Targeted species can vary from amphibians, birds to various mammals, dependent on the virus. For many mosquito-borne flaviviruses the spectrum of targeted species is incompletely understood, in particular with respect to their contribution to the maintenance of virus in certain geographical regions. Furthermore, little is known about virus and host factors contributing to species tropism. The present study utilized human and porcine monocyte-derived dendritic cells (MoDC) as a cell culture model to better understand Flavivirus species tropism and innate immune responses. MoDC were selected based on their presence in the skin and their role as an early target cell for several Flaviviruses and their role as immune sentinels. While differences in viral infectivity and replication were minor when comparing porcine with human MoDC for some of the tested Flaviviruses, a particularly strong replication in human MoDC was found with USUV, while JEV appeared to have a stronger tropism for porcine MoDC. With respect to innate immune responses we found high induction of TNF and IFN-β in both human and porcine MoDC after infection with JEV, WNV, and USUV, but not with DENV, ZIKV, and Wesselsbron virus. Spondweni virus induced these cytokine responses only in porcine MoDC. Overall, innate immune responses correlated with early infectivity and cytokine production. In conclusion, we demonstrate Flavivirus-dependent differences in the interaction with MoDC. These may play a role in pathogenesis but appear to only partially reflect the expected species tropism.

## Introduction

Within the genus of Flavivirus more than 60 species are described, which are mostly transmitted by arthropods to vertebrates. 50% of these virus species are mosquito-born, 28% are transmitted by ticks, and for the rest the vector is unknown (Simmonds et al., 2017). Some Flaviviruses such as Japanese encephalitis virus (JEV) infect a broad range of vertebrate hosts varying from amphibians, to birds and various mammals, while others have restricted host affinity, such as Dengue virus (DENV) only known to infect primates (Go et al., 2014; Simmonds et al., 2017). The majority of Flaviviruses are kept in enzootic cycles between hematophagous arthropods and vertebrates, vectors, which are infected during the blood sucking on viremic hosts. More than half of the described Flaviviruses cause zoonotic diseases, ranging from febrile illness, to encephalitis-related disease or life-threatening hemorrhagic fever (Borio et al., 2002; Weissenböck et al., 2010; Go et al., 2014; Gould et al., 2017). The fact that several Flaviviruses have recently emerged, such as West Nile virus (WNV) and Zika virus (ZIKV) in the Americas, and WNV and Usutu virus (USUV) in Europe represents a serious warning that the current distribution of Flaviviruses could expand in the near future (Gould et al., 2017). The recent identification of human cases of DENV after local transmission in different countries of Europe (France and Spain) highlights the risk of the introduction of new Flaviviruses where competent mosquito vectors are present, such as *Aedes (A.) albopictus*. It is important to note that none of the recent Flavivirus outbreaks have been predicted and that factors contributing to emergences are not well understood. For many of the less studied Flaviviruses the potential contribution of various vertebrate species to the maintenance of the virus is incompletely understood (Go et al., 2014). In particular, the susceptibility of animals living in close proximity to man such as domestic animals and livestock are important.

Despite the relatively broad host range, Flaviviruses have preferential vertebrate hosts, which differ in a virus-specific manner demonstrating adaptation to particular vertebrates. Examples are the preferences of JEV serocomplex viruses for birds, and in the case of JEV also for pigs, in which the virus causes several days of viremia, making the pig an amplifying host during epidemics (Turtle and Solomon, 2018). It is also important to note that dependent on the species the outcome of vertebrate infections ranges from inapparent infection to severe disease. For example, WNV and JEV can cause particularly severe disease in horses. Furthermore, particular vertebrates play an important role in maintaining the virus in the environment and therefore play a role as a “reservoir” (Kuno et al., 2017). Despite the veterinary and public health threat caused by these viruses the knowledge on the viral and host factors responsible for species tropism and the above-mentioned pathogenic features of Flavivirus infection is very limited. A relevant cell culture system modeling early events in infection, such as replication and innate immune responses would be very valuable to address the above questions. Cell lines are problematic because differences in their susceptibility to infection and support of viral replication often depend more on the different cell lines rather than the species of origin, as documented for ZIKV (Chan et al., 2016). This may be due to de-differentiation during their generation.

For such *in vitro* studies, the present work employed monocyte-derived dendritic cell (MoDC). This was based on the rational that these and related cells are present at the site of entry in the skin, where they are believed to support the virus replication of several Flaviviruses early after infection, and participate in early innate immune responses (Wu et al., 2000; McCullough et al., 2009; Schmid and Harris, 2014; Schmid et al., 2014; Hamel et al., 2015; Bowen et al., 2017; Vielle et al., 2018). MoDC cells can be generated from any species if sufficient numbers of blood monocytes can be obtained and recombinant cytokines are available. Another major advantage is that with animals of sufficient size blood sampling does not require killing. Furthermore, during infection-caused skin inflammation monocytes will be attracted and differentiate into both MoDC and monocyte-derived macrophages (Tamoutounour et al., 2013), which are likely to be involved in innate immune responses against arboviruses as demonstrated for DENV (Schmid and Harris, 2014; Schmid et al., 2014).

Considering the importance of pigs for livestock in many parts of the world and its known role as amplifying host for JEV, we decided to investigate if and how porcine MoDC differ from their human counterparts in their interaction with a collection of Flaviviruses. Our aim was on one side to evaluate how well the MoDC culture model can reflect species tropism of flaviviruses, and on the other side to identify differences between Flaviviruses in infection, replication and innate immune responses. To this end, we selected viruses that have caused recent epidemics including JEV, WNV, USUV, DENV, and ZIKV. We also included Spondweni virus (SPOV) and Wesselsbron virus (WESSV), both currently circulating only in Sub-Saharan countries. SPOV was included as a virus being closely related to ZIKV, and WESSV as a virus having a tropism for domestic animals, in particular ruminants (Hubalek et al., 2014b). Our data demonstrate Flavivirus-dependent species differences in virus susceptibility, replication and innate responses, and thereby provides information on species tropism of emerging Flaviviruses.

## Materials and Methods

### Ethics Statement

All procedures involving animals comply to the Animal Welfare Act (TSchG SR 455), the Animal Welfare Ordinance (TSchV SR 455.1), and the Animal Experimentation Ordinance (TVV SR 455.163) of Switzerland. All studies were reviewed by the ethical committee for animal experiments of the canton of Bern and approved by the cantonal veterinary authorities (Amt für Landwirtschaft und Natur LANAT, Veterinärdienst VeD, Bern, Switzerland). Specifically, porcine blood sampling was approved with the license #BE88/14. Human buffy coats were provided by the Swiss Transfusion SRC (Swiss Red Cross) Inc. (Regional transfusion blood service, Bern, Switzerland) which collected blood from anonymized healthy donors after ethical approval, and authorized use of human buffy coats given by the Swiss Transfusion SRC Institutional review board. All performed experiments were done following protocols designed according to the guidelines of the institution.

### Viruses

In this study we included different Flavivirus: JEV Laos strain (genotype 1; GenBank CNS769_Laos_2009; kindly provided by Prof. Remi Charrel, Aix-Marseille Université, Marseille, France), WNV NY99 (GenBank DQ211652.1; kindly donated by Prof. Martin Groschup, Friedrich-Loeffler-Institute, Germany), USUV SAAR-1776 strain (GenBank AY453412; kindly provided by Prof. Richard Hoop, University of Zürich, Zürich, Switzerland); DENV-3 VN32/96 strain, (serotype 3, GenBank EU482459, kindly provided by Dr. Katja Fink, Singapore Immunology Network, SIgN, Singapore), ZIKV strain PR-2015 (Asian lineage, PRVABC59; GenBank KX377337; obtained from Public Health England PHE); SPOV strain SM-6 V-1s (South Africa, GenBank DQ 859064.1 Originator: Oxford Institute of Virology, provided by EVAg, Marseille, France) and WESSV strain SAH-177 99871-2, passage 8 (South Africa, GenBank EU707555.1, Originator: UTMB collection, provided by EVAg, Marseille, France). All Flaviviruses were propagated in *A. albopictus* C6/36 cells (ATCC^®^ CRL-1660™) in MEM (Gibco, Lucerne, Switzerland) supplemented with sodium pyruvate at 100 mM (Gibco), non-essential amino acids (MEM NEAA; Gibco) and 2% fetal bovine serum (FBS) (*v/v*) (Biochrome, Bioswisstec AG, Schaffhausen, Switzerland) at 28°C and in 5% CO_2_ atmosphere conditions. Virus titrations were obtained using C6/36 cells as previously described (Ricklin et al., 2016). Infected cells were detected using immunoperoxidase monolayer assay (IPMA) with the anti-flavivirus E mAb 4G2 (ATCC, HB-112™). Titers were calculated and expressed as 50% tissue culture infective dose per ml (TCID_50_/ml).

### Porcine and Human Monocyte-Derived Dendritic Cells Differentiation

Porcine and human MoDC (pMoDC and hMoDC, respectively) were generated as previously described (Carrasco et al., 2001; Vielle et al., 2018). Briefly, porcine blood was collected from specific pathogen free (SPF) Swiss Large White pigs breed in our own facilities. Then peripheral blood mononuclear cells (PBMC) were isolated using ficoll-paque density centrifugation (1.077 g/L; GE Healthcare Life Sciences, Dübendorf, Switzerland). Human PBMC were isolated from buffy coats of anonymous healthy blood donors (Interregional blood transfusion SRC Ltd, Bern) by the same procedure described for porcine cells. Porcine monocytes were sorted as CD172a^+^ cells using monoclonal antibody (mAb), clone 74-22-15A (hybridoma kindly provided by Dr. A. Saalmüller, Veterinary University of Vienna, Austria) and anti-Mouse IgG MicroBeads (Miltenyi Biotec, Germany). Human monocytes were sorted as CD14^+^ cells using coated magnetic beads (human) according to the manufacturer's instructions (Miltenyi Biotec). For both species, we employed LS magnetic columns and the MACS system (Miltenyi Biotec). Then porcine monocytes were cultured at 5 × 10^5^ cell/ml in Dulbecco's modified Eagle's medium (DMEM) containing Glutamax (ThermoFisher Scientific, Zug, Switzerland) supplemented with 10% of FBS (Gibco), porcine GM-CSF (Summerfield et al., 2003) and porcine IL-4 (100 U/ml, own production; Carrasco et al., 2001). Similarly, human monocytes were plated at 1 × 10^6^ cells/ml in RPMI 1640 (Gibco) supplemented with 10% FBS (Gibco), Glutamax (Gibco), penicillin-streptomycin (Gibco), human GM-CSF (100 ng/ml; Miltenyi Biotec), and human IL-4 (40 ng/ml; Miltenyi Biotec). Then, cells were incubated for 6 days at 39°C (for porcine cells) or 37°C (for the human cells) and 5% CO_2_; on the third day of incubation fresh medium supplemented with cytokines was added to the cultures. MoDC differentiation was verified by microscopy and by flow cytometry. The culture resulted in over 95% DC-like cells defined as for porcine cells CD172a^+^CD80/86^+^ and CD14^low^ expression (Carrasco et al., 2001) and as CD11c^+^CD14^−^CD19^−^CD3^−^CD56^−^ cells (human) (Vielle et al., 2018).

### Flavivirus Infection on pMoDC and hMoDC

Porcine MoDC and hMoDC were incubated for 1.5 h at 39 or 37°C (for pMoDC or hMoDC, respectively) in 5% CO_2_ with the virus preparations at a multiplicity of infection (MOI) of 1 TCID_50_ per cell. Then, the cells were washed 3 times with phosphate buffered saline (PBS), and fresh medium supplemented with 2% FBS and cytokines as described above were added to the cells. After 24 and 48 h post infection (hpi), supernatants were harvested and stored at −80°C. MoDC were harvested as cell suspensions with cold PBS/5xEDTA and fixed with 4% (*w/v*) paraformaldehyde (PFA) for 10 min at room temperature, then washed with PBS 0.1% (*w/v*) saponin, and immunolabelled with mAb 4G2 in 0.3% (*w/v*) saponin in PBS during 15 min on ice. After washing the cells with 0.1% (*w/v*) saponin in PBS, anti-mouse IgG2a conjugated with Alexa 647 fluorochrome (ThermoFisher) was added for 10 min on ice. The cells were acquired on a FACSCantoII (Becton Dickinson) and results analyzed with Flowjo V.9.1 software (Treestar, Inc., Ashland, OR, USA). For analyses doublets and cells with low forward/side scatter corresponding to debris and dead MoDC were excluded by electronic gating. For the quantification of dead cells in the cultures we also employed FSC/SSC gating previously shown to correspond to dead MoDC detected by propidium iodide staining (Garcia-Nicolas et al., 2014). Viral titers were determined in C6/36 cells for JEV, WNV, USUV, ZIKV, DENV, SPOV, and WESSV using IPMA as described above.

### Cytokines Measurement

The porcine TNF and IL-1β, and the human TNF, IL-1β, and IFN-β expressions were quantified using commercial kits (R&D Systems, UK), with detection limits of 30 pg/ml for porcine and human TNF kit, 60 or 4 pg/ml for the porcine or human IL-1β ELISA, respectively, and 10 pg/ml for the human IFN-β test. Porcine IFN-β production was measured with reagents from Kingfisher with all incubations done at room temperature. Briefly, the polyclonal capture antibody against porcine IFN-β was diluted at 1 μg/ml to coat ELISA plates overnight at room temperature, then blocked with 4% FBS in PBS (also used as diluent for the other ELISA components) for 2 h. Samples (1:1 in diluent) and standards (2-fold-dilutions from 500 to 2 pg/ml of provided porcine IFN-β) were added at a final volume of 100 μl/well and incubated for another 2 h. After washing, the biotinylated detection antibody (0.5 μg/ml) was added for 2 h followed by Streptavidin-coupled horseradish peroxidase (DAKO) for 30 min, and the final addition of 3,3′,5,5′-tetramethylbenzidine (TMB; Dako) for the colorimetric reaction. After 30 min incubation in the dark, the reaction was stopped with 50 μl of 0.18 M sulfuric acid solution and absorbance was measured at 450 nm.

### Statistics

Figures and data analyses employed GraphPad Prism 7 Software (GraphPad Software, Inc., San Diego, CA, USA). All experiments were independently performed between 3 and 9 times with cells from different donors, and each condition in triplicates. For viral titrations, differences between groups were assessed using a Kruskal–Wallis test, and for individual differences the Mann–Whitney *U*-test with Bonferroni correction as *post hoc* was employed. For group differences in the percentages of infected cells and levels of cytokines expression comparisons, we employed a one-way ANOVA test with Bonferroni correction as *post hoc*. Correlation analysis between infected cells, dead MoDC, viral titers, and expressed cytokines were calculated by Spearman's Rho analysis; a correlation between two different factors was considered relevant with R^2^ > 0.5. For all data a *p* value lower than 0.05 was considered statistically significant. In the Figures ^*^ indicates *p* ≤ 0.05, ^**^p ≤ 0.002, ^***^p ≤ 0.001 and ^****^p ≤ 0.0001.

## Results

### hMoDC and pMoDC Differ in Their Susceptibility to Flaviviruses

**Figure 1:**
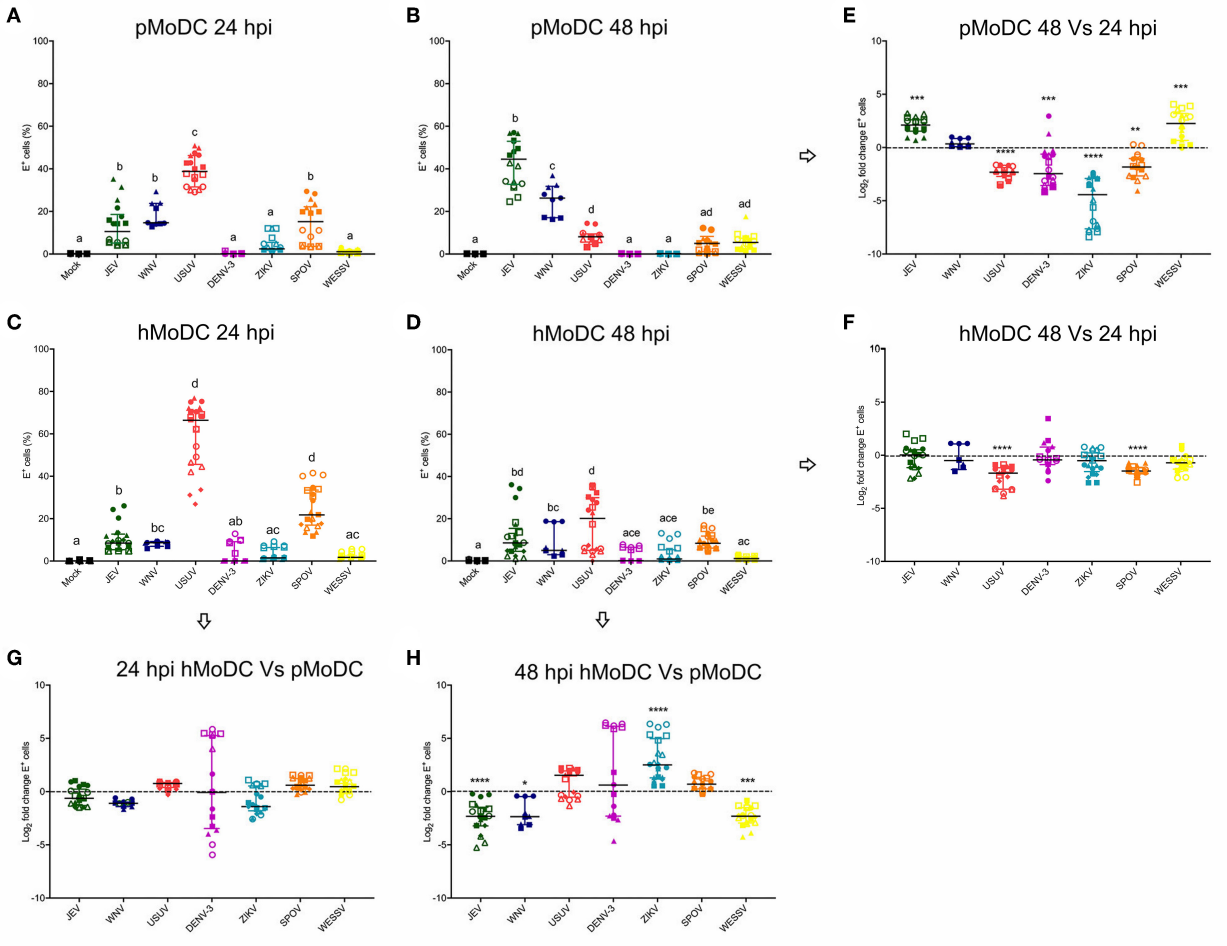
Comparative susceptibility of pMoDC and hMoDC to selected Flaviviruses. **(A–D)** E protein expression in pMoDC and hMoDC after infection with different viruses at a MOI of 0.1 TCID_50_ per cell. E protein expression was quantified after 24 **(A,C)** and 48 hpi **(B,D)** by flow cytometry. **(G,H)** Relative ability of Flaviviruses to infect hMoDC and pMoDC shown as fold change of infection (E protein positive cells) calculated at 24 **(G)** and 48 **(H)** hpi. **(E,F)** Fold change in infected cells between 24 and 48 hpi, shown for pMoDC and hMoDC, respectively. All experiments were performed in triplicates and repeated at least three, and up to seven times with cells from different donors. Each symbol represents a different blood donor. Results are presented as scatter plots showing the mean and all points. The different superscript letters in **(A–D)** indicate significant differences (*p* ≤ 0.05) between the different viruses. Fold change infected cells results are expressed in logarithmic scale of base 2; for each infection condition significant differences between the calculated fold change and the reference level (equal to 0, dotted line) are indicated (^*^p ≤ 0.05; ^**^p ≤ 0.002; ^***^p ≤ 0.001; ^****^p ≤ 0.0001).

With the aim to investigate differences in the susceptibility of pMoDC and hMoDC to different Flaviviruses, the percentages of E protein expression were determined at 24 and 48 hpi (Figure 1). At 24 h pMoDC showed the highest susceptibility to USUV followed by JEV, WNV and SPOV, and the lowest infectivity was found with DENV-3 and ZIKV (Figure 1A). With hMoDC, highest susceptibilities were found with USUV and SPOV, followed by JEV, WNV, DENV-3, ZIKV, and WESSV (Figure 1C). Interestingly, for none of the viruses a statistically significant difference was found when infectivity was compared at 24 h (Figure 1C). At 48 h, infectivity in pMoDC was significantly increased for JEV and WESSV, but decreased for USUV, DENV-3, ZIKV, and SPOV (Figures 1B,E). This contrasted to hMoDC, in which for none of the viruses an increased infectivity was found. USUV and SPOV cause reduced levels of infection (Figures 1C,D). When the percentages of infected cells from different hosts were compared at 48 hpi, we observed species-dependent differences in the Flavivirus infectivity. JEV, WNV, and WESSV showed a higher preference for porcine cells, while ZIKV for hMoDC (Figure 1H).

### Species-Dependent Differences in Flavivirus Replication in MoDC

**Figure 2:**
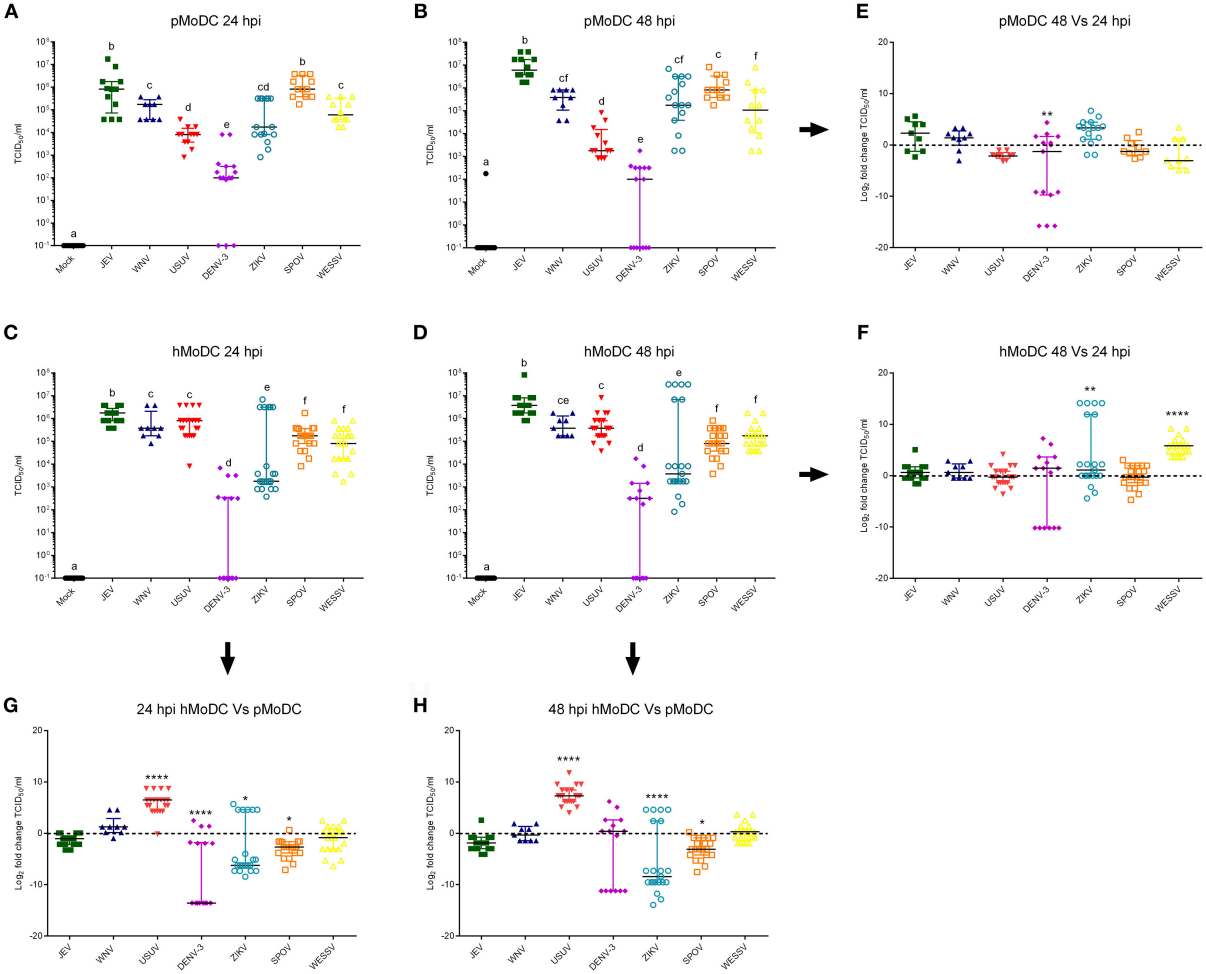
Flavivirus shedding after infection of pMoDC and hMoDC. **(A–D)** Viral titers released from pMoDC and hMoDC after infection with the different Flaviviruses at a MOI of 0.1 TCID_50_ per cell. Supernatants were collected at 24 **(A,C)** and 48 hpi **(D,B)**. All Flaviviruses titers were quantified using in C6/36 cells. **(G,H)** Relative ability of the viruses to replicate in human and porcine MoDC, shown as fold change of titers at 24 **(G)** and 48 **(H)** hpi. **(E,F)** Ratio of viral titers from at 48 vs. 24hpi for pMoDC and hMoDC, respectively. All experiments were performed in triplicates and repeated at least three, and up to seven times with cells from different donors. Each symbol represents a different blood donor. Results are presented as scatter plots showing the mean and all points. The different superscript letters in **(A–D)** indicate significant differences (*p* ≤ 0.05) between the different viruses. Fold change infected cells results are expressed in a logarithmic scale of base 2; for each infection condition significant differences between the calculated fold change and the reference level (equal to 0, dotted line) are indicated (^*^p ≤ 0.05; ^**^p ≤ 0.002; ^***^p ≤ 0.001; ^****^p ≤ 0.0001).

Titrations of the supernatants from the experiments shown in Figure 1 collected at 24 hpi, demonstrated that in pMoDC JEV and SPOV replicated to the highest titers, followed by WESSV, WNV, ZIKV, USUV, and finally DENV-3 (Figure 2A). In hMoDC at 24 h, high titers were found for JEV, WNV, USUV followed by SPOV, WESSV, ZIKV, and DENV-3 having again the lowest titers (Figure 2C). Species comparison at 24 h revealed higher titers of USUV but significantly lower levels of DENV-3, ZIKV, and SPOV when comparing hMoDC to pMoDC (Figure 2G). At 48 hpi in pMoDC, virus titers remained without significant changes with the exception of decreasing DENV-3 titers (Figures 2B,E). For hMoDC we found an increase for ZIKV and WESSV titers over time (Figures 2D,F). The species comparison of viral titers at 48 hpi revealed that again USUV had a clear advantage for replication in hMoDC. Surprisingly, SPOV and ZIKV better replicated in porcine cells (Figure 2H).

### MoDC Death Induced by Flaviviruses

**Figure 3:**
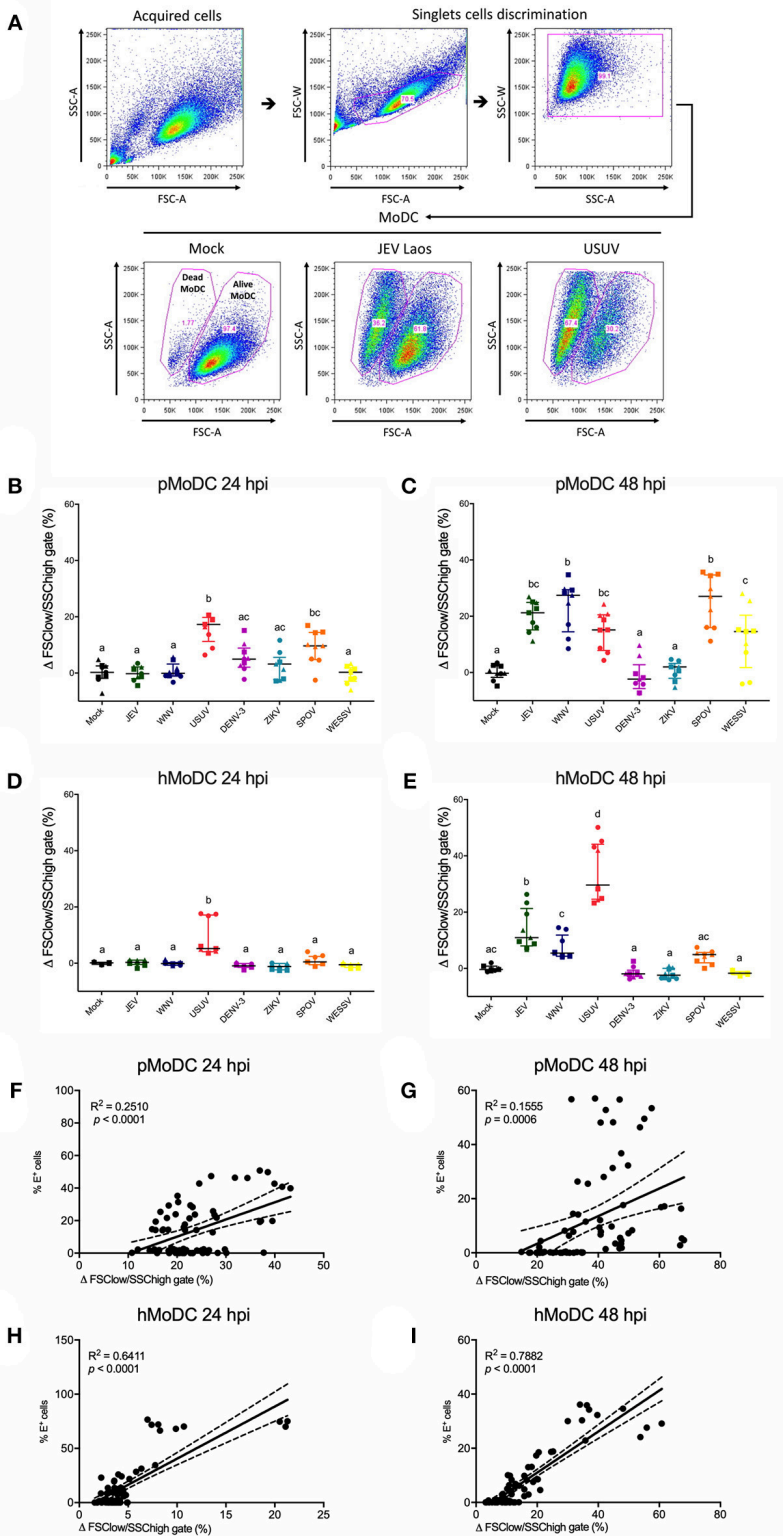
Flavivirus induced cell death in pMoDC and hMoDC. MoDC were infected with different Flaviviruses at MOI of 0.1 TCID_50_ per cell. **(A)** Gating strategy to eliminate doublets and quantify shrunken dead MoDC based on their forward/side scatter plots. Representative results are shown for Mock, JEV, and USUV. **(B–E)** Difference between the percentage of the average of shrunken in the mock with the virus-challenged cells was calculated and represented as Δ Dead MoDC (%) and represented at 24 and 48 hpi for pMoDC (**B,C**, respectively) and hMoDC (**D,E**, respectively). Each symbol represents a different blood donor. Results are presented as scatter plots showing the mean and all points. Different superscript letters indicate significant difference (*p* ≤ 0.05) between the percentages of dead MoDC induced by distinct viruses. **(F–I)** Correlation analysis between the number of dead cells and E positive cells at 24 and 48 hpi for pMoDC **(F,G)** d hMoDC **(H,I)** calculated by Spearman's Rho analysis. Correlations are shown as linear regression, R^2^ and *p* values are indicated for each analysis.

We next determined the impact of Flavivirus infection on MoDC viability. To this end we employed forward/side scatter plots, to determine the percentages of shrunken cells with increased granularity as shown in Figure 3A. At 24 hpi, pMoDC death was limited but significantly induced following challenge with USUV, SPOV followed by ZIKV and DENV (Figure 3B). In hMoDC, also low levels of dead cells were found at the early time point with significantly higher death induced by USUV (Figure 3D). This dramatically changed at 48 hpi when Flavivirus infection was associated with induction of death in pMoDC by most of the tested viruses with the exceptions of DENV-3 and ZIKV (Figure 3C). At 48 hpi in hMoDC, USUV infection induced the highest levels of cell death followed by JEV and WNV (Figure 3E). Interestingly, the correlation between cell death and infection (E protein expression) was higher for hMoDC when compared to pMoDC (Figures 3F–I).

### Innate Immune Responses in MoDC

**Figure 4:**
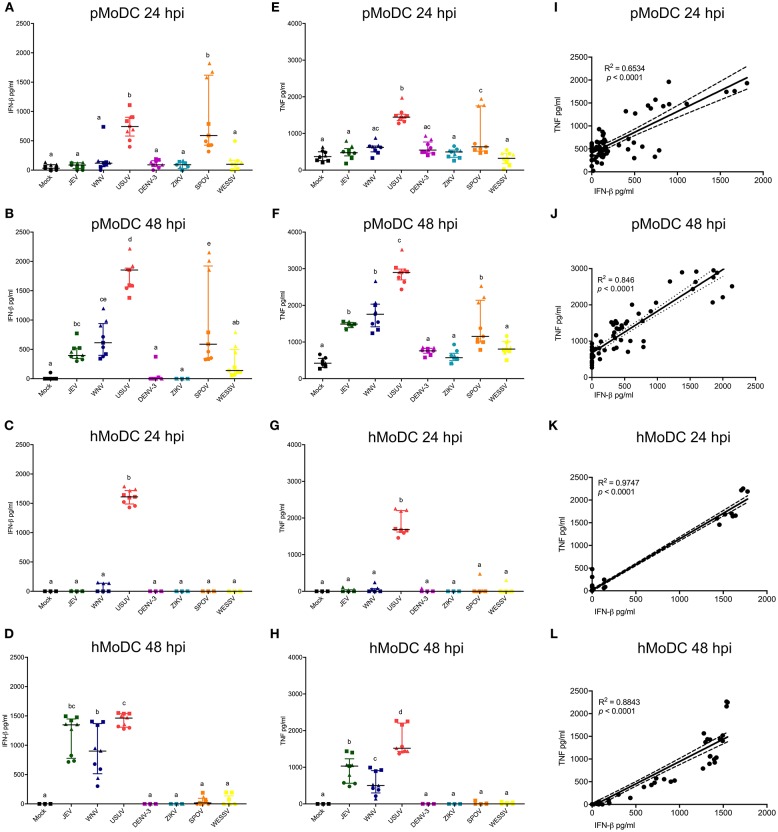
Cytokine responses following infection of MoDC. **(A,B)** IFN-β levels in supernatants of pMoDC harvested at 24 and 48 hpi (**A,B**, respectively). **(C,D)** IFN-β levels in supernatants of hMoDC harvested at 24 and 48 hpi (**C,D**, respectively). **(E,F)** TNF levels in supernatants of pMoDC harvested at 24 and 48 hpi (**A,B**, respectively). **(G,H)** TNF levels in supernatants of hMoDC harvested at 24 and 48 hpi (**C,D**, respectively). Supernatants were derived from the cultures shown in Figures 1, 2. TNF was quantified by ELISA. Each symbol represents a different blood donor. Results are presented as scatter plots showing the mean and all points. Different superscript letters indicate significant differences (*p* ≤ 0.05) between the amount of TNF induced in MoDC by distinct Flaviviruses. **(I,J)** Correlation between IFN-β and TNF for pMoDC at 24 and 48 hpi. **(K,J)** Correlation between IFN-β and TNF for hMoDC at 24 and 48 hpi. For **I–L**, correlations were calculated by Spearman's Rho analysis and are shown as linear regression, R^2^ and *p-*values are indicated for each analysis.

These analyses focused on IFN-β, TNF, or IL-1β secretion by MoDC. At none of the tested time points, we were able to detect virus-induced IL-1β with pMoDC and hMoDC. Overall, USUV induced higher secretion of IFN-β compared to the other viruses in both species and at any investigated time points (Figures 4A–D). Interestingly, in pMoDC SPOV induced strong IFN-β secretion (Figures 4A,B). In hMoDC, although at 24 hpi only USUV induced IFN-β, at 48 hpi also JEV and WNV induced significant levels of IFN-β secretion (Figures 4C,D). Interestingly, ZIKV and DENV never induced any IFN-β secretion in MoDC (Figures 4A–D).

Concerning TNF responses, similar results were obtained as for IFN-β. Both USUV and SPOV induced TNF production in porcine cells at 24 hpi. At 48 h, this cytokine was induced after challenge with JEV, WNV, USUV, and SPOV in pMoDC (Figures 4E,F). TNF responses in hMoDC were only observed with USUV at 24 h, and at 48 h after infection with the closely related viruses of the JEV serocomplex (JEV, WNV, and USUV; Figures 4G,H).

Considering the similarity of the IFN-β and TNF results we tested the correlation of these two parameters. Indeed, we found a strong correlation between the secretion of IFN-β and TNF in cells from both species at the tested time points (Figures 4H–L), indicating that both cytokines are triggered by similar innate pathways.

### Correlation of Innate Immune Responses With Infection

**Figure 5:**
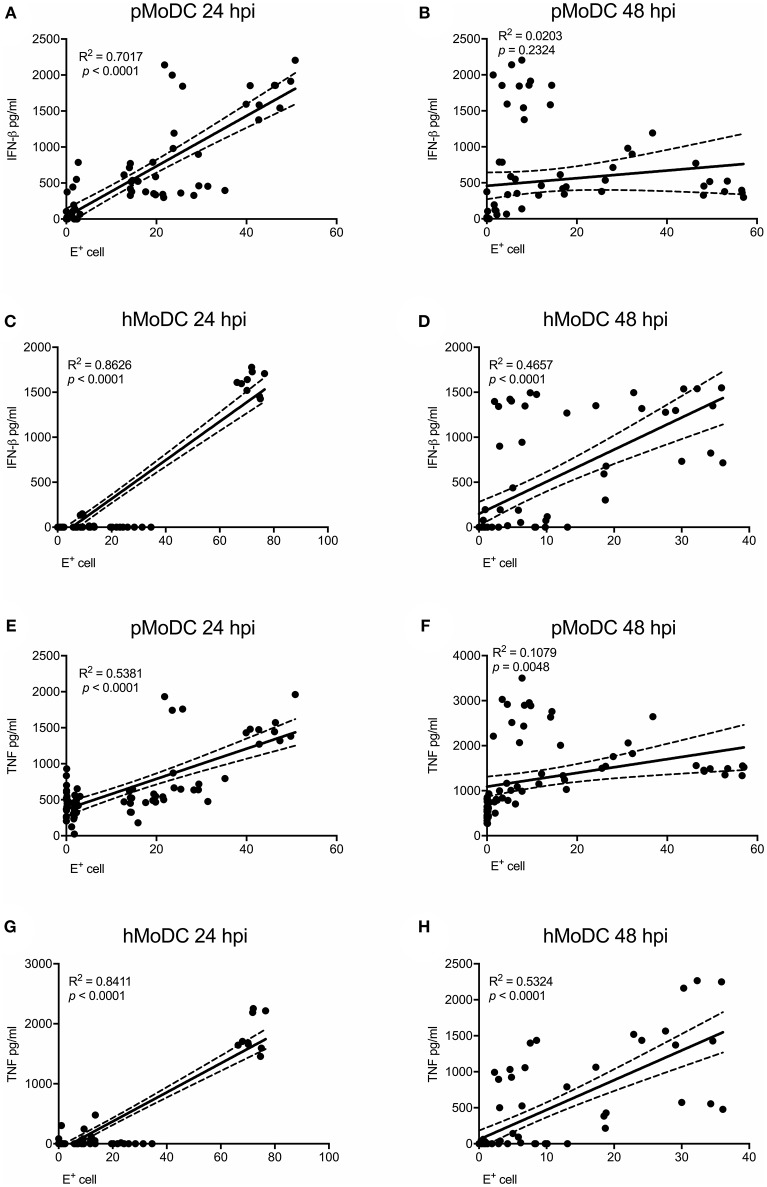
Correlation analysis between E protein positive cells and pro-inflammatory cytokines. Correlations between E protein positive cells and IFN-β **(A–D)** or TNF **(E–H)** for pMoDC at 24 and 48 hpi (**A,E** and **B,F**; respectively) and for hMoDC at 24 and 48 hpi (**C,G** and **D,H**; respectively) were calculated by Spearman's Rho analysis. Correlations are shown as linear regression, R^2^ and *p* values are indicated for each analysis.

As IFN-β and TNF are presumably induced by viral RNA, we analyzed the correlations of IFN-β and TNF to the percentage of virus-infected cells and also viral titers at the time of supernatant harvest. At 24 hpi the percentage of virus-infected pMoDC highly correlated with the secretion of IFN-β and TNF (R^2^ values of 0.7 and 0.53, respectively), but not at 48 hpi (Figures 5A,B,E,F). With hMoDC we found a positive correlation for both time points between the number of infected cells and the secretion of cytokines (Figures 5C,D,G,H). On the other hand, cytokines secretion did not correlate with the viral titers for any of the selected host species, indicating a lack of association between both factors (Supplementary Figure 1). We also tested a possible association cell death and IFN-β/TNF secretion. While this correlation between was weak for pMoDC, a higher positive correlation was found for hMoDC (Supplementary Figure 2). Altogether, these results suggest that innate immune responses depend on the number of infected cells. In general, only viruses inducing relatively high levels of infection cause a detectable cytopathic effects. Importantly, for the viral titers we found neither a positive nor negative correlation with cytokine responses.

## Discussion

Considering the dual host life with alternation between arthropods and vertebrates requiring adaptation to quite different hosts, it is not surprising that many mosquito-borne Flaviviruses, such as WNV and JEV can infect a wide range of vertebrate hosts. Nevertheless, there are clearly preferential vertebrate hosts, which differ in a viruses-specific manner demonstrating adaptation to particular vertebrates. The present study was initiated, considering that the knowledge on virus adaptation is incomplete in terms of the degree of adaptation of some of the newly emerged and some of the more locally restricted and neglected Flavivirus to particular vertebrate species is unclear. Our aim was to identify virological and immunological factors involved using MoDC as *in vitro* model. To validate the model, we selected human and pigs. Altogether, our data demonstrate prominent differences in the rate of infection, released infectious virus and innate immune responses, which were dependent on both the host and the virus species. Considering this, the crucial question is how well do these differences recapitulate what is known about species tropism. For JEV we observed that pMoDC were more permissive and replicated to the highest titers at 48 h compared to the rest of the selected Flaviviruses, which appears to be in accordance with an enormous amount of field and *in vivo* experimental data on pigs (Mansfield et al., 2017). Also, hMoDC supported high levels of JEV replication in accordance to work published by others (Cao et al., 2011; Gupta et al., 2014). The high seroconversion rate in endemic areas indicates that humans are indeed highly susceptible to JEV, although only few develop the disease. Nevertheless, human do not develop viremia of sufficient duration and intensity to re-infect feeding mosquitoes (Turtle and Solomon, 2018). A possible explanation could be that the strong IFN-β response identified in MoDC would limit further virus replication and dissemination in most infected individuals. Of note, it has been speculated that in those patients developing encephalitis, infected monocytic cells could be the Trojan horse mediating transport of the virus across the blood-brain barrier (Lannes et al., 2017).

For WNV we found that despite of low numbers of WNV-infected pMoDC, the virus efficiently replicated. Although some *in vivo* experiments suggest that pigs would not play an important role as amplifying host for WNV (Ilkal et al., 1994; Teehee et al., 2005), a seroconversion of pigs has been demonstrated in about of 22.5% of tested serum samples from feral pigs in the US, in 17.6% of wild boars in Europe and in 15.5% of farmed pigs also in Europe (Gibbs et al., 2006; Escribano-Romero et al., 2015). This clearly demonstrated that pigs are susceptible to WNV and a role of this species in the epidemiology cannot be excluded. Interestingly, our study showed that WNV infected and replicated to high titers in hMoDC without inducing early innate cytokine responses. These results which support previously published data (Rawle et al., 2015) could indicate that WNV has undergone a certain degree of adaptation to human cells.

During the last two decades, since USUV emerged in Europe, several outbreaks occurred affecting wild and domestic birds (Weissenböck et al., 2003; Hubalek et al., 2014a; Grottola et al., 2017; Michel et al., 2018). Our *in vitro* results demonstrate that although at 24 h many pMoDC are infected by USUV, the infection rate drops strongly at 48 h and gives rise only to relatively low viral titers. This is associated with high IFN-β and TNF responses, possibly indicating a poor adaptation of this virus to the pig. This would be in line with surveillance data for WNV and USUV. Although antibodies against both viruses were found in wild boars, in domestic pigs only WNV-specific antibodies were detected (Escribano-Romero et al., 2015). However, it is not known if wild boars differ from domestic pigs in their susceptibility to USUV. hMoDC were also highly susceptible to USUV but replicated to higher titers when compared to pMoDC. Nevertheless, similar to another report (Cacciotti et al., 2015), also in hMoDC high IFN-β and TNF responses were found which would be expected to limit virus spread *in vivo*. Field data demonstrate that humans are infected by USUV in endemic areas, although only in rare cases with severe clinical symptoms or even fatal outcome (Pecorari et al., 2009; Santini et al., 2015; Grottola et al., 2017).

The present study showed that pMoDC are resistant to infection by DENV-3, which is likely to be responsible for the lack of innate immune responses in pMoDC. These results are in line with previous field observations, supporting that DENV vertebrate hosts are mainly restricted to primates (Chen and Vasilakis, 2011). Nevertheless, some authors suggested that other vertebrates could be hosts for DENV in the rainforest, or that pigs could be used as an animal model (de Thoisy et al., 2004; Cassetti et al., 2010). With human cells, we observed a low susceptibility to infection by DENV-3, and an overall inefficient virus replication. This could be related to the strain used as previous reports indicate highly variable levels of infection which may depend on the serotype and virulence (Navarro-Sanchez et al., 2003; Silveira et al., 2011).

With respect to ZIKV, although the frequency of E protein expressing pMoDC was low, porcine cells supported ZIKV replication well, in absence of any detectable IFN-β and TNF response. These *in vitro* data could be in line with experimental studies showing that newborn piglets developed viremia between 3 and 5 days after intra-dermal inoculation with ZIKV (Darbellay et al., 2017a,b). In accordance to previously published work, our data show that ZIKV replicates in hMoDC in a donor dependent manner and that this infection did not induce effective innate immune responses (Bowen et al., 2017; Vielle et al., 2018).

Although SPOV is genetically closely related to ZIKV (Haddow et al., 2016), pMoDC were more susceptible to the infection by SPOV than ZIKV and the virus efficiently replicated in swine cells. The infection caused high amounts of IFN-β and TNF, possibly limiting prolonged virus infection. With respect to possible hosts that could maintain or amplify SPOV in the wildlife, previous reports demonstrated that neither birds nor rodents seem to act as reservoir for the virus, and experimental studies suggest non-human primates as possible target for the virus (Haddow et al., 2016). Our results suggest that serological field data from endemic areas should be performed to address a possible role of pigs in the viral life cycle. In hMoDC, SPOV showed comparable characteristics in terms of its efficient replication and the lack of IFN-β and TNF induction.

A considerable broad range of hosts have been described for WESSV, including human, sheep, goat, cattle, pigs, dogs, rats, and other wild life species (Coetzer and Barnard, 1977; Simpson et al., 1979; Coetzer and Theodoridis, 1982; Barnard, 1997; Hubalek et al., 2014b; Diagne et al., 2017). However, in neither human nor porcine MoDC we found high levels of infected cells but relatively good viral titers in the supernatants of the cell cultures, without evidence for induction of innate immune responses.

Another potentially interesting readout was virus induced cell death, which correlated well with the levels of infected cells, indicating a direct viral cytopathogenic effect. Nevertheless, we also found a high correlation of cell death with IFN-β and TNF responses. This may indicate that host response to infection may contribute to cell death.

Our results showing IFN-β and TNF following certain Flavivirus infections, are in accordance with previously published works indicating that hMoDC produce both TNF and IFN type I secretion during the infection by DENV or JEV (Sooryanarain et al., 2012; Schmid et al., 2014). Interestingly, in our *in vivo* experimental studies in pigs we could neither detected IFN-β nor TNF in serum samples (Ricklin et al., 2016; Garcia-Nicolas et al., 2017). This would suggest that the production of such cytokines by MoDC would be locally restricted. Porcine MoDC, secrete lower levels of IFN-β after JEV challenge than hMoDC, which is possibly due to a delayed exposure of JEV dsRNA in the cytosolic compartment of porcine MoDC as previously reported (Espada-Murao and Morita, 2011). The observation that certain of the selected Flaviviruses such as ZIKV, SPOV, and WESSV did not induce innate immune responses despite good replication in hMoDC, indicates differences in the viruses' ability to evade innate immunity and raises the questions about the mechanisms. For instance, in ZIKV-infected cells the existence of subgenomic Flavivirus RNA has been related to the blocking of IFN type I transcription induced by RIG-I (Manokaran et al., 2015; Villordo et al., 2016). For other Flaviviruses, such as JEV, DENV or ZIKV, NS2A, NS4A, NS4B or NS5 proteins were demonstrated to participate in evasion of the IFN type I system (Miorin et al., 2017). Clearly, future studies are required to investigate how these different mechanisms of innate immune evasion contribute to the species tropism of distinct viruses.

In addition to differences in innate immune responses, the role of viral receptors for species tropism requires consideration. A difficulty could be the relative flexibility of *Flaviviruses* in receptor usage. For example, this can be glycosaminoglycans like heparan sulfate (Su et al., 2001), vimentin (Liang et al., 2011), laminin (Thongtan et al., 2012), CD4 (Thongtan et al., 2012), α5β3 integrins (Chu and Ng, 2004), CD209, and CLEC4G (Shimojima et al., 2014; Wang et al., 2016) for attachment of JEV to host cells.

When interpreting the results of the present work, it is important to note that for logistic reasons we were only able to include one strain for each virus species, and extrapolations to all viruses from the studied species should be done with care as strain differences can be important as mentioned above for DENV. Furthermore, in most cases it is impossible to directly correlate our *in vitro* data to *in vivo* experimental data. These of course are completely lacking for humans and are only partially available for pigs. Nevertheless, altogether some clear differences in Flavivirus-MoDC interactions were identified *in vitro*, indicating that such data can give valuable information on virus-host interactions. These can model certain aspects of *in vivo* infection, and can help to evaluate differences in innate immune responses against Flaviviruses. In this context, the biggest surprise was the high level of infection and replication of USUV in hMoDC, which may be interpreted as a warning concerning the zoonotic potential of USUV.

## Data Availability

All datasets for this study are included in the manuscript. Raw flow cytometry and ELISA data supporting the conclusions of this manuscript will be made available by the authors, without undue reservation, to any qualified researcher.

## Author Contributions

OG-N, MR, and AS: conceptualization; OG-N, ML, MR, and AS: methodology; OG-N, ML, and MR: investigation; OG-N and AS: formal analysis; AS: supervision; OG-N and AS: writing–original draft; OG-N, ML, MR, and AS: writing–review and editing; AS: funding acquisition.

### Conflict of Interest Statement

The authors declare that the research was conducted in the absence of any commercial or financial relationships that could be construed as a potential conflict of interest.
