# Corrigendum: Monocyte-Derived Dendritic Cells as Model to Evaluate Species Tropism of Mosquito-Borne Flaviviruses

**DOI:** 10.3389/fcimb.2019.00163

**Published:** 2019-05-15

**Authors:** Obdulio García-Nicolás, Marta Lewandowska, Meret E. Ricklin, Artur Summerfield

**Affiliations:** ^1^Institute of Virology and Immunology (IVI), Bern, Switzerland; ^2^Department of Infectious Diseases and Pathobiology, Vetsuisse Faculty, University of Bern, Bern, Switzerland; ^3^Graduate School for Cellular and Biomedical Sciences, University of Bern, Bern, Switzerland; ^4^Department of Emergency Medicine, Inselspital, University Hospital Bern, Bern, Switzerland

**Keywords:** Flavivirus, monocyte-derived dendritic cells, *in vitro* model, infection, tropism, innate immune response

In the original article, there was a mistake in Figure 1. The plots **C** and **F** in Figure 1 were wrong and not from hMoDC as they should have been. The corrected Figure 1 appears below. The text of the Figure legends and the article remains unchanged as they describe the correct Figure 1. The authors apologize for this error and state that this does not change the scientific conclusions of the article in any way. The original article has been updated.

**Figure 1:**
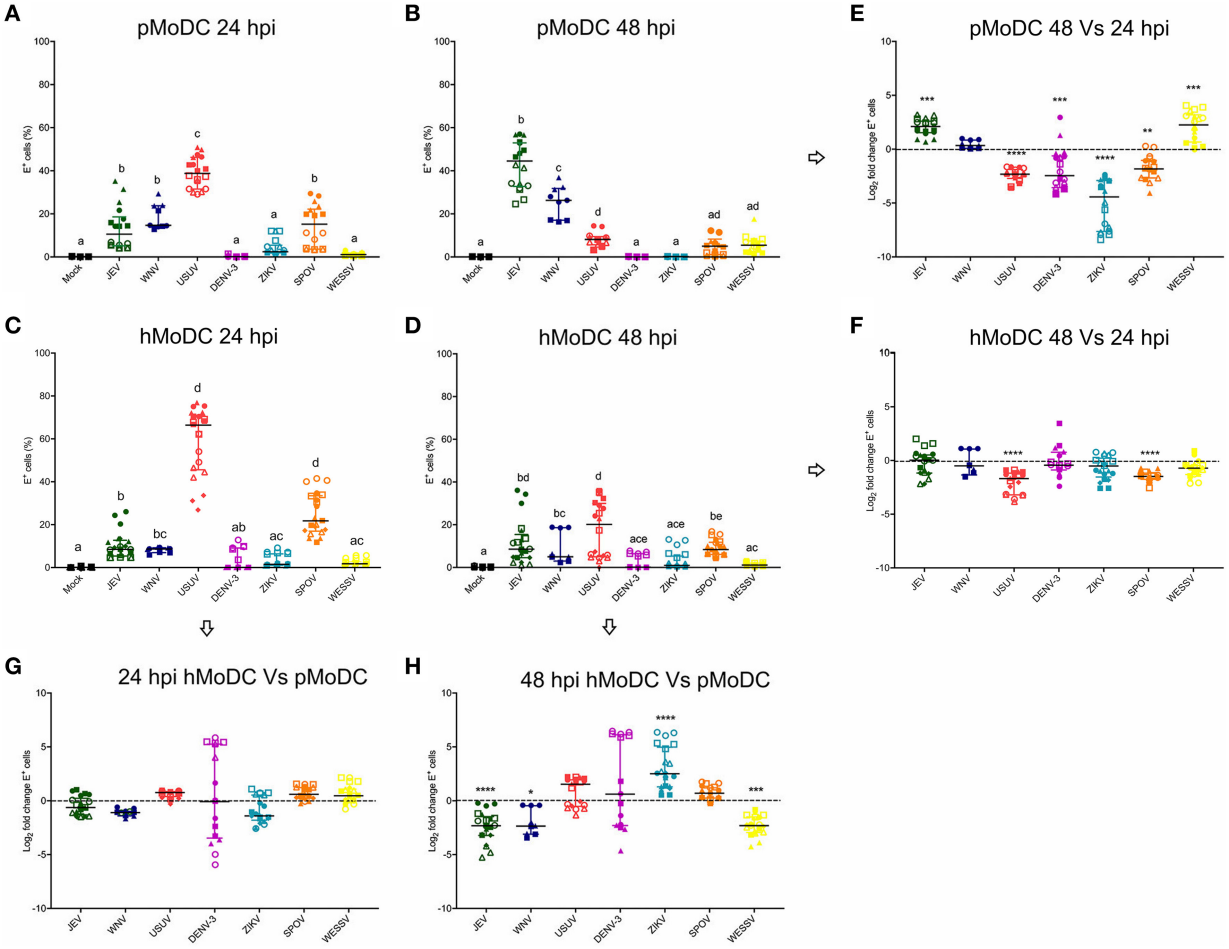
Comparative susceptibility of pMoDC and hMoDC to selected Flaviviruses. **(A–D)** E protein expression in pMoDC and hMoDC after infection with different viruses at a MOI of 0.1 TCID_50_ per cell. E protein expression was quantified after 24 **(A,C)** and 48 hpi **(B,D)** by flow cytometry. **(G,H)** Relative ability of Flaviviruses to infect hMoDC and pMoDC shown as fold change of infection (E protein positive cells) calculated at 24 **(G)** and 48 **(H)** hpi. **(E,F)** Fold change in infected cells between 24 and 48 hpi, shown for pMoDC and hMoDC, respectively. All experiments were performed in triplicates and repeated at least three, and up to seven times with cells from different donors. Each symbol represents a different blood donor. Results are presented as scatter plots showing the mean and all points. The different superscript letters in **(A–D)** indicate significant differences (*p* ≤ 0.05) between the different viruses. Fold change infected cells results are expressed in logarithmic scale of base 2; for each infection condition significant differences between the calculated fold change and the reference level (equal to 0, dotted line) are indicated (^*^p ≤ 0.05; **p ≤ 0.002; ***p ≤ 0.001; ****p ≤ 0.0001).

