# Supplementary material for: Monocyte-Derived Dendritic Cells as Model to Evaluate Species Tropism of Mosquito-Borne Flaviviruses
Source: Front Cell Infect Microbiol. 2019 Jan 28;9:5. doi: 10.3389/fcimb.2019.00005 (PMC6360178; doi:10.3389/fcimb.2019.00005)
Supplement: Supplementary file 1 [file Data_Sheet_1.PDF]

Supplementary Material

Monocyte-derived dendritic cells as model to evaluate species tropism of mosquito-borne flaviviruses

García-Nicolás O., Lewandowska M., Ricklin M.,E., Summerfield A.

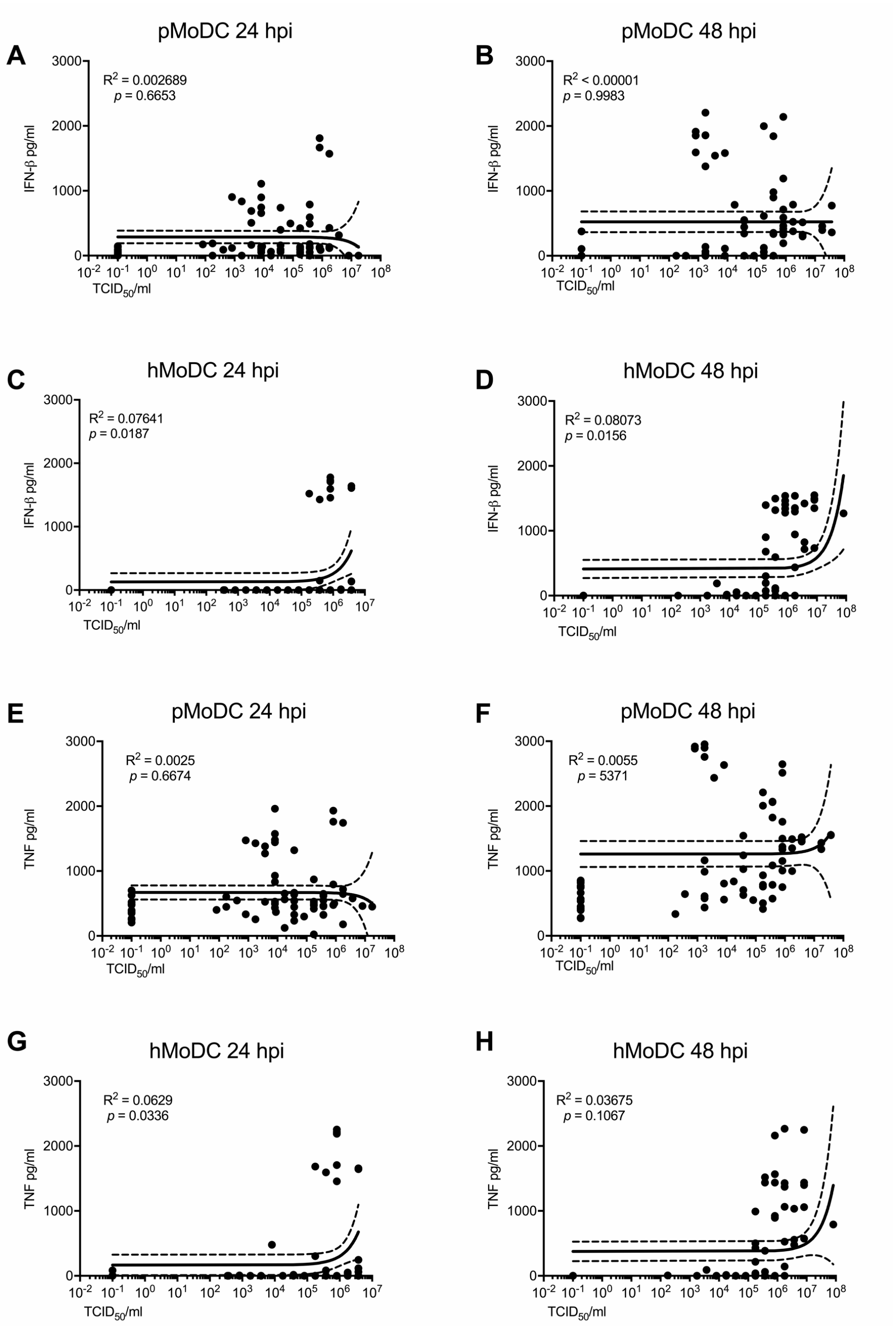

Sup 1. Correlation analysis between virus titers and cytokines. Correlations between virus titers and IFN-β (A-D) or TNF (E-H) for pMoDC at 24 and 48hpi (A, E and B, F; respectively) and for hMoDC at 24 and 48hpi (C, G and D, H; respectively) were calculated by Spearman's Rho analysis. Correlations are shown as linear regression, R^2 and p values are indicated for each analysis.

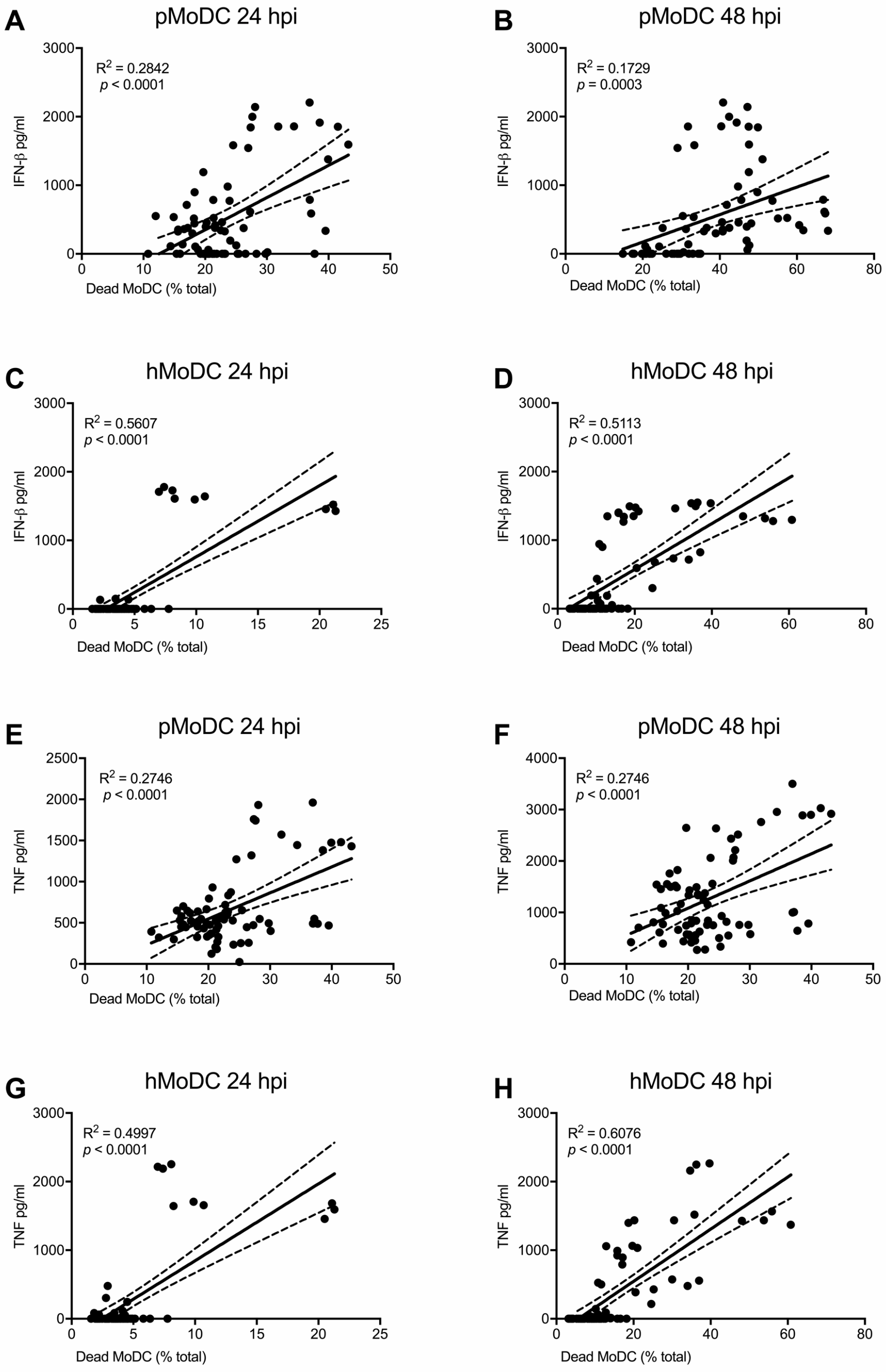

Sup 2. Correlation analysis between percentage of dead MoDC and cytokines. Correlation between the percentage of dead cells and IFN-β (A-D) or TNF (E-H) for pMoDC at 24 and 48hpi (A, E and B, F; respectively) and for hMoDC at 24 and 48hpi (C, G and D, H; respectively) were calculated by Spearman's Rho analysis. Correlations are shown as linear regression, R^2 and p values are indicated for each analysis.
